# Update on the epidemiology of obstructive sleep apnea syndrome in Central Eastern Tunisia over 11 years

**DOI:** 10.11604/pamj.2024.47.206.42949

**Published:** 2024-04-24

**Authors:** Rania Bouchech, Maissa Ben Jmeaa, Mouna Baklouti, Gammoudi Nouha, Sourour Yaich, Ghazi Sakly, Ridha Ben Cheikh

**Affiliations:** 1 Sleep Unit, Neurophysiology Department, Sahloul Hospital, Sousse, Tunisia,; 2 Community Health and Epidemiology Department, Hedi Chaker University Hospital, Sfax, Tunisia

**Keywords:** Obstructive sleep apnea syndrome, trends, incidence, obesity, severity, gender, age

## Abstract

**Introduction:**

obstructive sleep apnea syndrome (OSAS) is the most common sleep-related breathing disorder. Knowledge about OSAS incidence trends could be extremely useful in assessing health needs and implementing preventive measures accordingly. This study aimed at the epidemiological and clinical specificities of OSAS and to give an update on its current chronological trends.

**Methods:**

we conducted a retrospective study including all cases of OSAS diagnosed over 11 years, from January 1, 2012, to December 31, 2022, at the Sleep Unit of the Neurophysiology Department of the Sahloul University Hospital, Tunisia.

**Results:**

overall, 848 new cases of OSAS were diagnosed. The mean annual number of OSAS cases was 74.8/year. The crude incidence rate (CIR) was 12.3/100000 inhabitants/year, it was significantly increasing over the years (rho=0.7; p=0.01). The median age was 56 (IQR= [48-64]) years, it increased significantly during the study period from 54 years (IQR= [43-63]) in 2012 to 58 years (IQR= [50.5-65]) in 2022 (rho=0.7; p=0.015). The median BMI was 35.5 (IQR= [31.3-40.3]) kg/m^2^. The median BMI of patients diagnosed with OSAS increased significantly from 34.6 kg/m^2^ to 38.3 kg/m^2^ (rho=0.75; p=0.008). This equated to an annual increase in median BMI of 0.41 kg/m^2^. The median AHI showed a significant upward trend for all patients, rising from 26.30 events/h in 2012 to 34.07 events/h in 2022 (rho=0.68; p=0.02).

**Conclusion:**

the CIR of OSAS is related to BMI and age. Thus, we assume that it will continue to increase in the coming years with the rise in obesity and the aging of the population.

## Introduction

Sleep disorders encompass a wide spectrum of diseases with significant individual health consequences and high economic costs to society [1]. Obstructive sleep apnea syndrome (OSAS) is the most common sleep-related breathing disorder [1] defined as a repeated collapse of the upper airway during sleep that results from obstructive apneas and hypopneas [2]. It remains a major cause of morbidity and mortality worldwide, with more than 100 million people affected by the OSAS in 2007 [3], and with prevalence exceeding 50% in some countries [4]. Moreover, it is considered an independent risk factor for all-cause mortality with almost 2 times higher risk of death and 2.65 times increased risk of cardiovascular mortality [5]. As with other sleep disorders, OSAS has a broad impact on individuals. It affects their daily behaviors, cognitive abilities, and performance.

Besides, it exposes them to an increased risk of accidents, mood disorders, cancer, cardiovascular disease, and hypertension. Thus, early recognition and effective diagnosis of OSAS are needed to minimize its negative health impacts and improve daily functioning. Furthermore, knowledge about OSAS incidence trends can be invaluable to assess health needs and implement preventive measures accordingly. Because of the lack of exhaustive and updated data on OSAS in our region, we aimed to study the epidemiological and clinical specificities of OSAS in Central Eastern Tunisia and to give an update on its current chronological trends from 2012 to 2022.

## Methods

**Study design:** we conducted a retrospective study including all OSAS cases diagnosed over 11 years, from January the 1^st^ 2012 to December the 31^st^ 2022 at the Sleep Unit of the Neurophysiology Department, Sahloul University Hospital, Central East of Tunisia.

**Settings, participants, data collection sources, and inclusion and exclusion criteria:** data was retrospectively collected from the existing medical records of all OSAS cases diagnosed during the study period. It was an exhaustive study, in which every new patient affected by OSAS and that diagnosis was made during the study period was included in our study. At enrollment, relapsing patients were excluded to calculate the incidence rates. We collected data from patients´ clinical records. The database variables included patients´ socio-demographic characteristics in particular age and gender. Clinical features, such as the patient's medical history, body mass index (BMI), and clinical presentation were recorded.

**Variables:** according to BMI, patients were classified into five categories: underweight (BMI < 18.5 kg/m^2^), normal weight (BMI 18.5-24.9 kg/m^2^), overweight (BMI 25.0-29.9 kg/m^2^), obesity class I (BMI 30.0-34.9 kg/m^2^), obesity class II (BMI 35.0-39.9 kg/m^2^), extreme obesity class III (BMI≥40.0 kg/m^2^) [6]. Depending on the American Association of Sleep Medicine guidelines, the diagnosis of OSAS was confirmed by polysomnography (PSG) when the apnea hypopnea index (AHI) was 5 or greater events/h accompanied by daytime sleepiness. This kind of sleepiness was evaluated by the Epworth sleepiness scale. The OSAS severity was graded according to the AHI: mild OSAS with AHI of 5 to <15 events/h, moderate with AHI of 15 to 30 events/h, and severe with AHI of >30 events/h [7].

**Obstructive sleep apnea syndrome incidence rates:** according to the Tunisian National Institute of Statistics data, eligible patients were divided into 3 age categories: under 15 years, between 15 and 59 years, and 60 years and above. The crude incidence rate (CIR) was calculated in the three age groups and both genders and was revealed as numbers/100 000 population/year [8].

**Statistical methods:** statistical analysis was performed using SPSS.20 software. Categorical variables were referred to as numbers and percentages. The Kolmogorov-Smirnov test was used to assess the distribution of quantitative variables. Their results were presented as means ± standard deviation (SD) if they were normally distributed. Median and interquartile ranges (IQR) were used for non-normally distributed data. To compare two frequencies, we used the Chi-square test in independent samples. We calculated the correlation coefficient of Spearman (Rho) to analyze chronological trends over time. The odds ratio (OR) was used to measure the association between an exposure and an outcome. The simple linear regression was used to assess the relationship between two quantitative variables. The difference between the groups was considered significant when p<0.05.

**Ethical consideration:** this study did not have any intervention or experimentation on human beings and the participation was after patients´ consent. Anonymity was guaranteed and maintained.

## Results

**Patients´ characteristics:** over the 11-year study period, we recorded a total of 823 new cases of OSAS. The sex ratio (male/female) was 1.3. The average age at enrolment was 56 years (IQR = [48-64]) years and it increased significantly during the study period from 54 years (IQR = [43-63]) in 2012 to 58 years (IQR = [50.5-65]) in 2022 (rho = 0.7; p = 0.015). Of all patients eligible for our study, 498 cases (60.5%) were aged between 15 and 59 years. Comorbidities were found in 68.4% of patients (n=563). Cardiovascular (82.1%) and endocrine diseases (45.1%) were the most prevalent (Table 1). The median BMI was 35.5 (IQR = [31.3-40.3]) kg/m^2^. According to the BMI classification, 68.8%, 13.6%, and 0.2% of patients were obese, overweight, and underweight/normal, respectively. Nocturnal snoring was present in 97.3% of cases (n=801) with a mean duration of 4 (IQR = [2-6]) years. The median Epworth Sleepiness Scale was 13 (IQR = [9-16.2]). The daytime sleepiness was present in 68.7% of cases (n=565) (Table 1).

**Table 1:** socio-demographic, anthropometric, and clinical characteristics of patients with obstructive sleep apnea syndrome

Variables	Number	Percentage (%)
**Total**	823	100
**Gender**		
Males	462	56.1
Females	361	43.9
**Age groups (years)**		
<15	5	0.6
15-60	498	60.5
≥60	320	38.9
**Associated comorbidities**		
None	260	31.6
One or more	563	68.4
Cardiovascular diseases	462	82.1
Endocrine diseases	254	45.1
Respiratory diseases	54	9.6
Neurological disorders	47	8.3
**BMI (kg/m^2^)**		
< 18.5	1	0.1
18.5-24.9	8	1
25.0-29.9	112	13.6
30.0-34.9	196	23.8
35.0-39.9	183	22.2
≥40.0	188	22.8
Missing data	135	16.4
**Nocturnal snoring**		
Yes	801	97.3
No	22	2.7
**Day time sleepiness**		
Yes	565	68.7
No	258	31.3
**The severity of the obstructive sleep apnea syndrome**		
Severe	461	56
Moderate	134	16.3
Mild	228	27.7

**Figure 1:**
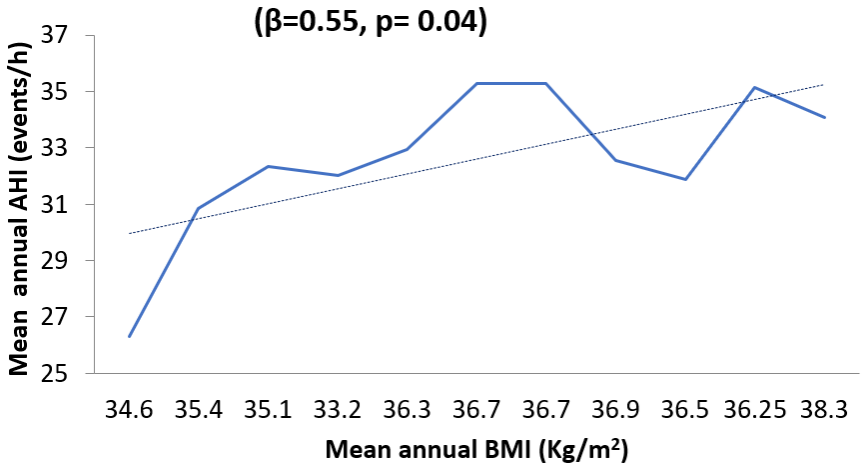
relationship of the mean annual apnea-hypopnea index and body mass index, 2012-2022

**Obstructive sleep apnea syndrome: epidemiology and clinical features:** the median AHI was 32.3 events/h (IQR = [14.1-51.2]). Severe OSAS accounted for 56% (n=461) and was markedly more common in males (OR=1.8; p<0.001), older patients (≥60 years) (OR=2.3; p<0.001), and obese patients (OR=2; p<0.001). Patients with associated cardiovascular diseases were 1.4 times (p=0.015) more likely to develop a severe OSAS. Over the study period, the median BMI of patients diagnosed with OSAS went up significantly from 34.6 kg/m^2^ to 38.3 kg/m^2^ (rho = 0.75; p = 0.008). This equated to an annual increase in median BMI of 0.41 kg/m^2^. Likewise, the median AHI had a major growing trend for all patients, rising from 26.30 events/h in 2012 to 34.07 events/h in 2022 (rho = 0.68; p = 0.02). The proportion of patients with severe OSAS considerably increased by 57.1% from 2012 (40.8%) to 2022 (64.1%) (rho = 0.8; p = 0.002). Simple linear regression analysis revealed that an increase in BMI by one unit was strongly associated with an increase in AHI of 0.55 events/h (Figure 1). There was a significant association between AHI and age, with an average increase in AHI of 0.25 events/h for every one-year increase in age (β = 0.25; p=0.003).

**Crude incidence rates of obstructive sleep apnea syndrome stratified by sex and age groups:** over the 11-year-study-period, the mean annual number of OSAS cases was 74.8/year and the CIR was 12.3/100 000 population/year. The sex-specific CIR of OSAS was 13.8/100 000 population/year in men and 10.7/100 000 population/year in women (Table 2). When stratified by age groups, the CIR was 52.7/100 000 population/year in elderly and 0.3/100 000 population/year in children (Table 2).

**Table 2:** mean annual number of new cases and crude incidence rates of obstructive sleep apnea syndrome stratified by gender and age groups

Variables	Average population	New cases	Mean annual number	Crude incidence rate^a^
**Total**	609615	823	74.8	12.3
**Gender**				
Males	303407	462	42	13.8
Females	306208	361	32.8	10.7
**Age groups (years)**				
<15	157869	5	0.4	0.3
15-60	396591	498	45.3	11.4
≥60	55155	320	29.1	52.7

a/100 000 population/year

**Figure 2:**
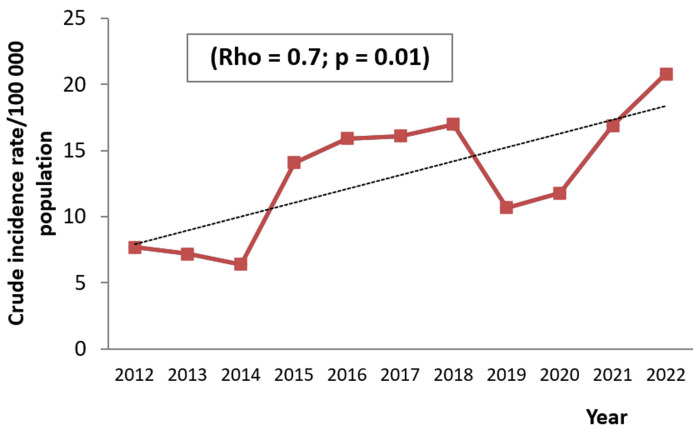
chronological trends of obstructive sleep apnea syndrome crude incidence rate between 2012 and 2022

**Figure 3:**
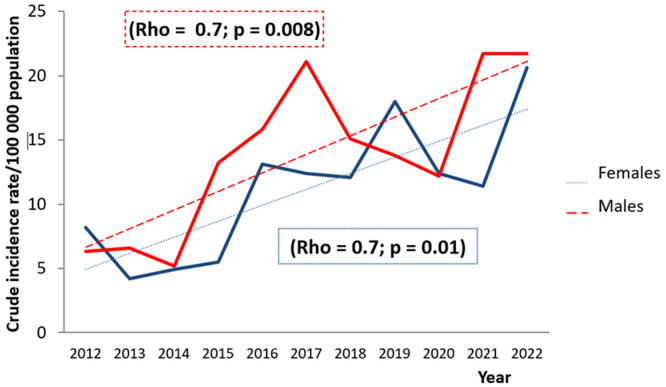
chronological trends analysis of obstructive sleep apnea syndrome crude incidence rates by gender between 2012 and 2022

**Figure 4:**
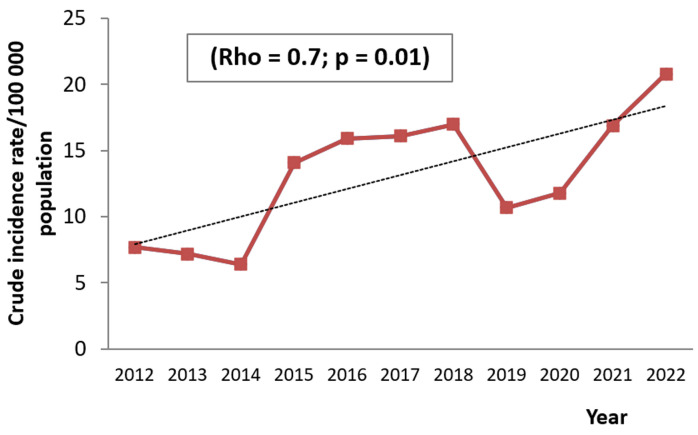
chronological trends analysis of obstructive sleep apnea syndrome crude incidence rates by age groups between 2012 and 2022

**Current trends in obstructive sleep apnea syndrome by sex and age groups:** the analysis of OSAS chronological trends demonstrated a significant increase in the CIR from 6.4/100000 population in 2012 to 20.8/100000 population in 2022, (rho = 0.7; p = 0.01). Yet, a decrease in the CIR was noted during the periods 2012-2014 and 2019-2021 (Figure 2). The gender-specific CIR of OSAS showed a significant increase for both females (rho = 0.7; p = 0.01) and males (rho = 0.7; p = 0.008) (Figure 3). Regarding trends analysis of OSAS incidence by age groups, patients aged 60 years old and above presented a significant augmentation between 2012 and 2022 (rho = 0.6; p = 0.03). The age groups 0-14 and 15-59 years old had an increasing trend, but the incidence rate variation trend was statistically not significant over time (Figure 4).

## Discussion

Our study showed an increasing number of sleep apnea cases during the study period, with the incidence of OSAS rising from 2012 to 2022. However, during the period between 2012 -2014 and 2019-2021, the occurrence has decreased. Over the period 2012-2014, we notice a decrease in incidence that predominates in women, who presented the major proportion of patients during this period. Women present atypical symptoms such as fatigue and asthenia leading to an under-diagnosis of the disease [9]. However, as shown by the various epidemiological studies and confirmed by our analysis, women did not represent a minority in sleep apnea syndrome.

In fact, according to the literature, 40% of patients referred to the sleep center for suspected apnea syndrome were women, which means that almost half of the consultants were female. The atypical clinical presentation needs to be better known by clinicians to overcome this bias. During the period 2019-2021, the decline of CIR could be attributed to covid19 pandemic. As a preventive measure, the number of polysomnography tests was reduced. Unlike these two periods, the increase in cases was expected as there was an increase in the prevalence of OSAS over the world. According to The Busselton study [10], the prevalence of sleep apnea rose from 26% in 1995 to 58% during the period 2010-2015. The proportion of women with sleep apnea rose from 19.5% to 39.2% over the period 1987-2007, which means that the rate had doubled over two decades according to another study [11].

The rising of new cases of sleep apnea in our study could be the result of an increase in the average age of our sample over the study period reflecting probably the aging of the Tunisian population. Indeed, the statistics about age structure showed that the Tunisian population aged over 65 rose from 7.06% to 9.2% from 2012 to 2022, which means during the study period, the increase in the proportion of this age group was about 2.14% [12]. It should be noted that studies conducted in a large number of cohorts had objectified a great prevalence of sleep apnea in elderly people, demonstrating that the prevalence of OSAS increased with age [11]. That might be explained by several mechanisms such as fat accumulation in the parapharyngeal region and alterations in the surrounding structure of the pharynx. Also, the increase in OSAS could be due to obesity, which is the main predisposing factor for OSAS [13]. As the worldwide obesity epidemic grows, the incidence and prevalence of sleep-disordered breathing are expected to increase in parallel. The Wiscon sleep cohort study indicated that a one-gap difference in body mass index was associated with a four-fold increase in the prevalence of the disease [14]. Other studies have shown that a 6-unit increase in BMI was associated with a fourfold increase in the risk of developing OSAS [15].

According to an Australian study, an annual increase in BMI of 0.61 units for men and 0.14 units for women was objectified in patients referred for suspected sleep apnea syndrome [16]. Similarly, a Canadian study showed an annual increase in BMI of 0.17 units for men and 0.34 units for women over almost a decade [17]. Therefore, obesity trends could be contributing to the increase in sleep apnea [17]. The associations between obesity and OSAS could be the outcome of multiple mechanisms, such as increased collapsibility of the upper airway and impaired neuromuscular control of upper airway patency caused by the local fat deposition [18].

Obesity could not only explain the increase in cases of OSA but also the worsening of the syndrome over time. In fact, according to the Wisconsin Sleep Cohort Study, weight change played a crucial role in the progression and regression of OSAS [15]. A long-term study of a middle-aged population with SAS [19] objectified that 10% weight gain was associated with a 32% increase in AHI. Young *et al*. estimated that 58% of moderate to severe cases of sleep apnea were associated with a BMI greater than 25 kg/m [18]. We can deduce that the high prevalence and the increase in AHI in our sample over time may reflect the growing severity of OSA in a population with a substantial prevalence of obesity. According to a similar study carried out in the region of Sousse in 2022 [20], the prevalence of obesity was approximately 28%.

However, this result may be explained by other hypotheses, such as the fact that clinicians referred polysomnography patients with highly suggestive symptomatology and whose clinical presentations were the most severe. However, the concomitant increase in AHI and BMI over time observed in our study suggested that OSAS was tending to become increasingly severe in our sample and that the increase in AHI was not the result of simple patient selection. The worsening of OSAS could also be a consequence of the increased incidence in the elderly population as older people were characterized by a more severe syndrome [10], but such conclusions should be interpreted with caution since the relationship between age and sleep apnea syndrome appears to be complex.

## Conclusion

To crown all, this study is the first to examine epidemiological trends in a clinical population of patients, with obstructive sleep apnea syndrome confirmed by polysomnography in Tunisia. Indeed, it is likely that the CIR of OSAS is related to BMI and age; thus, we assume that it will continue to increase in the coming years with the rise in obesity and the aging of the population. Our results are interesting as they provide valuable data for managing the causes of OSAS to reduce the associated healthcare costs and the negative impact of the disease. A management strategy needs to be developed not only by controlling the obesity rate but also by raising awareness among clinicians and by promoting sleep medicine.

### What is known about this topic

- *Obstructive sleep apnea syndrome: the most common sleep-related breathing disorder;*
- *Knowledge about obstructive sleep apnea syndrome incidence trends is valuable;*
- *Lack of exhaustive and updated data on obstructive sleep apnea syndrome in Central Eastern Tunisia*.

### What this study adds

- *An increasing number of sleep apnea cases over the years with a decreased occurrence has been in Central Eastern Tunisia;*
- *The CIR of OSAS is related to BMI and age;*
- *Urgent need to develop a management strategy not only by controlling the obesity rate but also by raising awareness among clinicians and by promoting sleep medicine*.
